# Tumor glycosylation and ABO phenotyping alterations in invasive urothelial carcinoma: A case report

**DOI:** 10.1016/j.htct.2026.108548

**Published:** 2026-10-09

**Authors:** Vanessa Cristina Fanger, Isabelle Carolina Cotrim Gozzi, Marina Cavalcanti de Albuquerque da Veiga Conrado, Regina Aparecida Cardoso, Vanderson Rocha, Alfredo Mendrone-Junior, Carla Luana Dinardo

**Affiliations:** a Fundação Pró-Sangue, Hemocentro de São Paulo, São Paulo, SP, Brazil; b Instituto de Ciências Biomédicas, Universidade de São Paulo, São Paulo, SP, Brazil; c Fundação Faculdade de Medicina da Universidade de São Paulo (FFMUSP), São Paulo, SP, Brazil

## Introduction

The ABO blood group system is widely recognized as a histo-blood group system, since its antigens are not restricted to erythrocytes but are also significantly expressed in a variety of epithelial tissues [1]. In the context of immunohematology and tumor biology, this characteristic is of particular relevance, as marked differences exist in carbohydrate profiles between normal and neoplastic tissues [1]. Malignant transformation promotes aberrant glycosylation patterns resulting from dysfunctional glycosyltransferase activity, leading to structural alterations of glycans expressed on the cell surface [2].

Similar to several other carbohydrate-based blood group systems, the biochemical identity of ABO antigens is determined by specific carbohydrate modifications at the terminal chains of cell membrane oligosaccharides [3]. Antigen A is characterized by the addition of N-acetylgalactosamine (GalNAc), whereas antigen B results from the incorporation of D-galactose. Both modifications occur on the precursor H antigen; the absence of enzymatic modification at this site defines the O phenotype. These antigenic determinants, which also include the Lewis system, are expressed on both glycoproteins and glycosphingolipids at the cell surface [2,3].

Beyond their classical expression on erythrocytes, ABO antigens are widely distributed in epithelial tissues of the respiratory and gastrointestinal systems and, particularly relevant, in the genitourinary tract. In these tissues, they play a fundamental role in processes such as cell adhesion, differentiation, and recognition [[1], [2], [3]]. Alterations in these glycobiological mechanisms have been extensively described in bladder carcinogenesis and represent one of the main biochemical changes associated with increased tumor invasiveness and metastatic potential [[1], [2], [3]].

In this context, acquired alterations in glycosylation patterns may generate challenging serologic discrepancies in oncology patients, resulting in loss or reduction of original ABO antigen expression as well as the emergence of paradoxical reactivities [2,3]. Such phenomena may mimic phenotypes distinct from those predicted by the individual’s genetic inheritance, reflecting cellular dedifferentiation, genomic instability, and enzymatic reprogramming characteristic of carcinogenesis [1]. Thus, ABO system expression acquires relevance not only from a biological perspective, but also in diagnostic and prognostic terms across different malignancies.

## Case report

A 72-year-old patient (AMMS) has been followed by the hematology team at Hospital das Clínicas since August 2015 due to a diagnosis of myelodysplastic syndrome with multilineage dysplasia. At the time of diagnosis, a trial of corticosteroid therapy was administered; however, due to the lack of clinical response, treatment with erythropoietin was subsequently initiated. Throughout the follow-up, between August 2015 and July 2021, immunohematologic testing demonstrated concordant forward and reverse ABO typing consistent with blood group O (Figure 1A).

**Figure 1:**
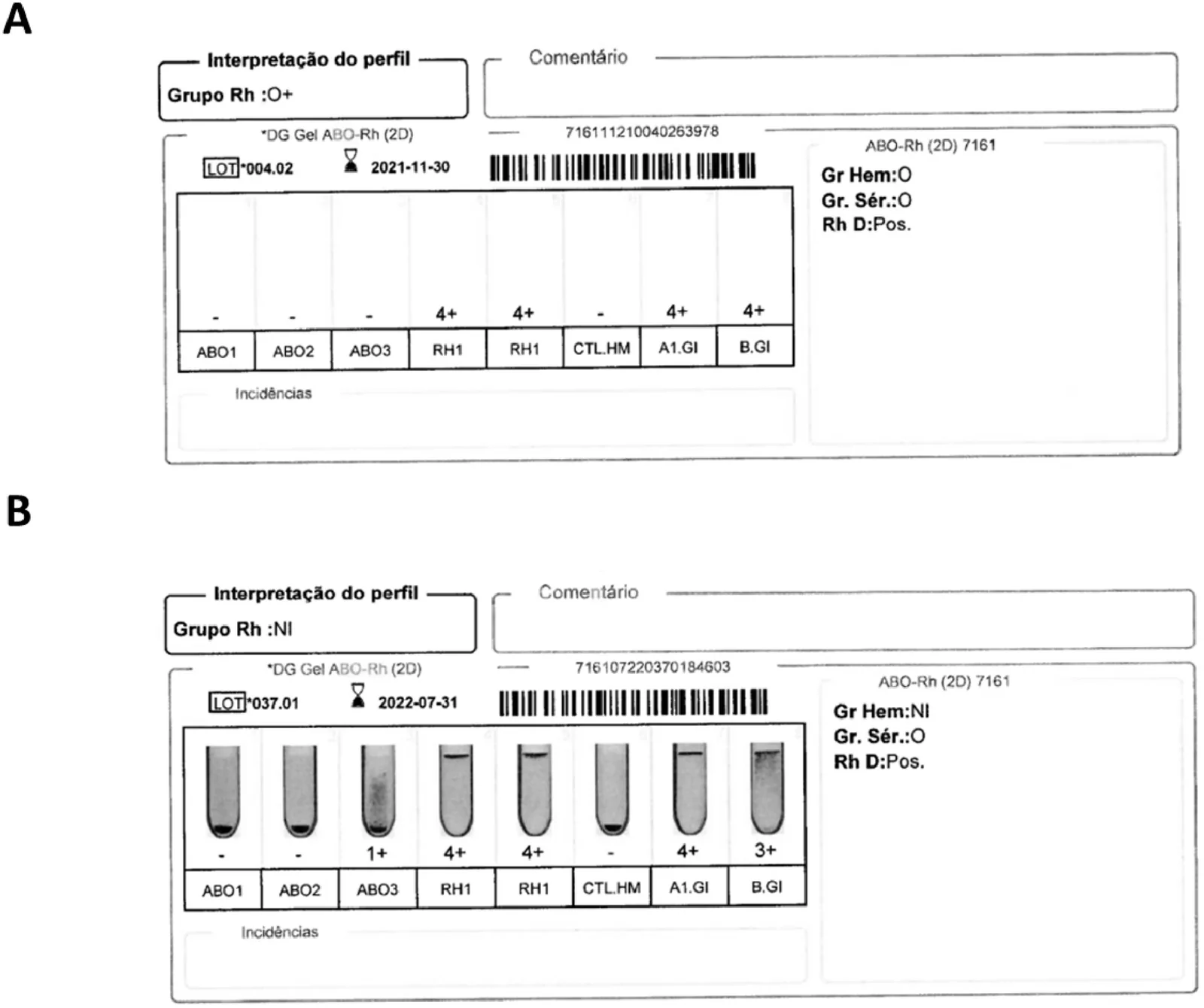
ABO–Rh typing performed using an automated gel microcolumn system. (A) The presence of agglutination in the Anti-A (A1.GI), Anti-B (B.GI), and Rh (RH1) wells indicates a positive reaction, consistent with blood group O. (B) The presence of agglutination in the ABO3 well (1+) represents a discrepancy relative to the absence of agglutination in the Anti-A (A1.GI) well.

In July 2021, after the onset of hematuria, invasive bladder carcinoma was diagnosed and initially treated with radiotherapy. From that point onward, serologic interference in ABO typing was observed, characterized by the presence of reactive anti-AB in forward typing (1+) (Figure 1B). The direct antiglobulin test (DAT) and antibody screening yielded negative results.

Forward typing was repeated by the tube method at room temperature and at 4 °C using an erythrocyte suspension in isotonic saline and commercial monoclonal antisera (Imunoscan), showing negative reactions with anti-A and anti-B. Enzymatic treatment of the red blood cell suspension with bromelain (Grifols) was also performed, with persistently negative results for anti-A and anti-B. The patient had no history of transfusion reactions and no laboratory findings suggestive of hemolysis.

Review of the transfusion history revealed that the patient had received, at another institution, apheresis platelets and buffy coat-derived platelet concentrates from donors of different ABO groups. Between March and July 2021, the patient received seven group O apheresis platelet concentrates, one group AB apheresis platelet concentrate, three group O pooled buffy coat-derived platelet concentrates, and three group O red blood cell concentrates at Fundação Pró-Sangue. Although the precise time interval from the last platelet transfusion performed at the external institution could not be determined, the immunohematologic discrepancy persisted over a prolonged period (from July 2021 to May 2022). This chronological persistence significantly reduces the likelihood of passive soluble antigen adsorption as the cause of the phenomenon.

On May 31, 2022, a new ABO typing was performed using gel card methodology (Grifols), which demonstrated a strongly reactive reverse grouping (4+) with A and B reagent red cells. In forward grouping, a weak reaction with anti-A (1+), no reaction with anti-B, and positivity with anti-AB (2+) were observed. Repeat testing by the tube method at room temperature using a 3% patient erythrocyte suspension showed negative anti-A and anti-B and positive anti-AB (2+). Tube testing at 4 °C yielded results concordant with those obtained by gel card. Reverse grouping was performed by gel card methodology using commercial 0.8% reagent A1 and A2 red cells (Grifols, Barcelona, Spain), both showing 4+ reactivity. Given the persistent serologic discrepancy, ABO genotyping was requested and identified the O1/O1 genotype.

## Discussion

The phenotypic transition observed from an originally group O typing to a weak A (1+) phenotype in an individual with an O1/O1 genotype highlights the impact of neoplasia on the erythrocyte glycocalyx. Loss or modification of ABH antigen expression in bladder tumors is a well-established marker of malignancy and a predictor of local invasion [4,5]. Among the hypothesized molecular mechanisms associated with this discrepancy are O-glycan truncation, leading to exposure of the Tn antigen (α- GalNAc) [2], and the proposed activity of the enzymes N-acetylglucosaminyltransferase I and III (GNT-I and GNT-III), which are responsible for the addition of N-acetylglucosamine (GlcNAc) residues to mannose residues of N-glycans [6]. Because the terminal A antigen also contains GalNAc as its immunodominant sugar, cross-reactivity with naturally occurring anti-A antibodies may occur, simulating an A subgroup phenotype in a genetically group O individual [2,3] (Figure 2).

**Figure 2:**
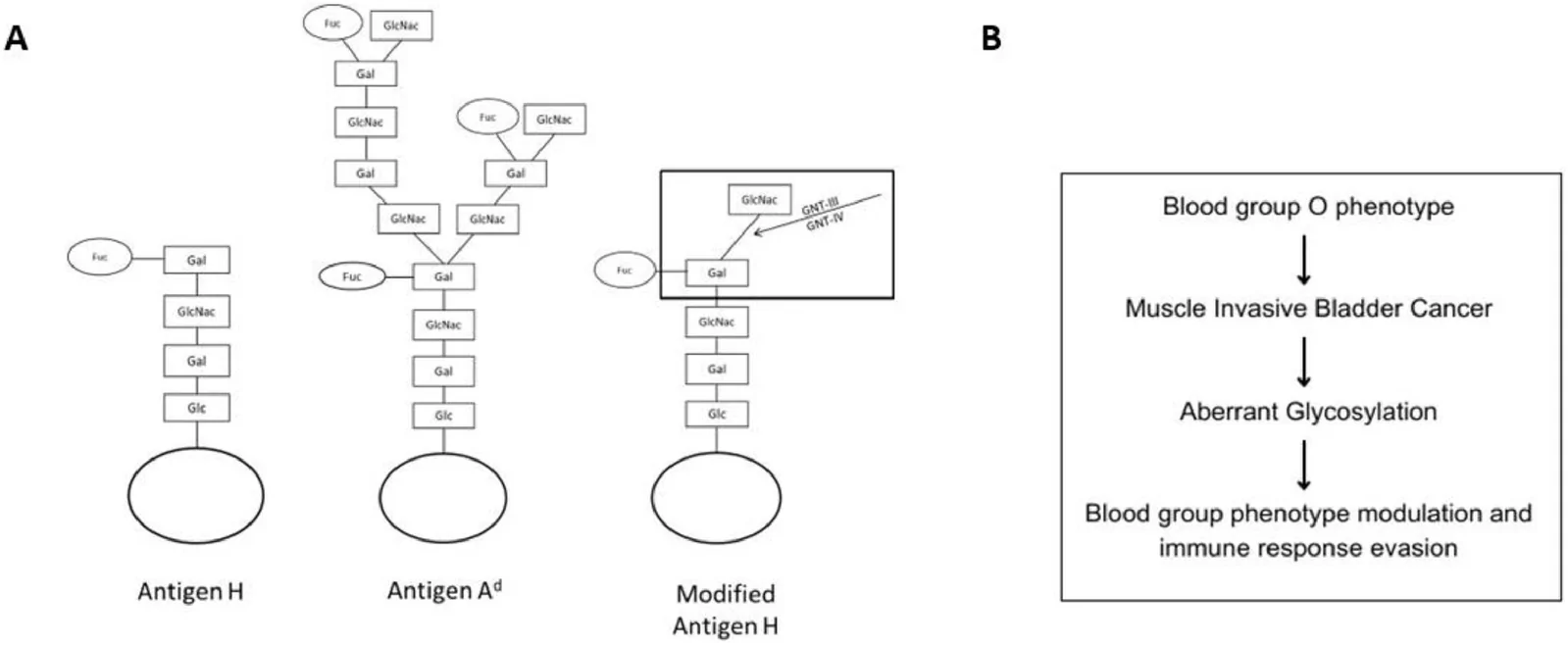
Schematic representations. (A) Carbohydrate structures of the H antigen, Aᵈ antigen, and the modified H antigen resulting from the activity of N-acetylglucosaminyltransferases I and III (GNT-I and GNT-III). (B) Proposed schematic model for blood group phenotype modulation in group O patients with muscle-invasive bladder cancer.

The clinical aggressiveness of the reported muscle-invasive carcinoma may also correlate with a possible overexpression of the enzyme C2GnT, which promotes the formation of core 2 O-glycans on the tumor cell surface. In theoretical models, these glycans enable binding of Galectin-3 to the MICA ligand, mechanically blocking tumor recognition by natural killer (NK) cells through the NKG2D receptor [5]. In addition, Galectins 1 and 3 can modulate immune responses by restricting T-cell receptor (TCR) signaling and promoting states of anergy and tolerance in T and B cells [7]. This mechanism of immune silencing is suggested to favor tumor cell survival in the circulation and enhance metastatic risk [2,4]. Furthermore, alterations in the glycosylation of integrin α3β1 and the formation of bisecting GlcNAc mediated by GNT-III modify cell adhesion, potentially facilitating tissue migration characteristic of the invasive phenotype [7,8].

Although alterations in the ABO system have historically been associated with leukemias [9], this case indicates that aggressive solid tumors might induce immunohematologic changes through tumor-specific molecular biology. While these comprehensive metabolic pathways remain to be experimentally confirmed in this specific case, they offer a plausible literature-based explanation for the observed serological phenomenon.

## Conclusion

In conclusion, this case demonstrates that ABO typing discrepancies in oncology can function as prolonged biochemical signatures of tumor progression and immune evasion. The persistent acquisition of an 'A-like' phenotype over several months highlights the role of tumor-induced aberrant glycosylation, potentially driven by pathways such as glycan truncation. In this scenario of neoplasia-induced phenotypic instability, molecular confirmation through ABO genotyping reaffirms its critical role as the gold standard to resolve complex serological discrepancies, ensuring accurate biological identification and ultimate transfusion safety.

## Funding

This study received no external funding.

## Conflicts of interest

The authors declare no conflicts of interest
